# Correction to: A novel strategy for efficient disaccharides synthesis from glucose by *β*-glucosidase

**DOI:** 10.1186/s40643-022-00535-1

**Published:** 2022-04-22

**Authors:** Kangle Niu, Zhengyao Liu, Yuhui Feng, Tianlong Gao, Zhenzhen Wang, Piaopiao Zhang, Zhiqiang Du, Daming Gao, Xu Fang

**Affiliations:** 1 State Key Laboratory of Microbial Technology, Shandong University, Qingdao, 266237 China; 2 Yantai Huakangrongzan Biotechnology Co., Ltd, Yantai, 264006 China; 3 Department of Rehabilitation Science, Graduate School of Health Science, Kobe University, Kobe, 6540142 Japan; 4 National Glycoengineering Research Center, Shandong University, Qingdao, 266237 China

## Correction to: Bioresour Bioprocess (2020) 7:45 https://doi.org/10.1186/s40643-020-00334-6

In the original publication of the article (Niu et al. 2020), the statement in the Funding information section was incorrectly given as ‘the National Key R&D Program of China (No. 2018YFA090010)’ and should have read ‘the National Key R&D Program of China (No. 2018YFA0901700)’. The original article has been corrected.
